# Micro-recovery dosing in elite sport: an initiative-taking, organization-wide recovery framework

**DOI:** 10.3389/fphys.2026.1951882

**Published:** 2026-09-15

**Authors:** Asaf Shalom, Roni Gottlieb, Albert Altarriba-Bartes, Franc García, Javier Espasa-Labrador, Javier Vilamitjana, Álvaro Velarde, Igor Jukic, Francesco Cuzzolin, Nicolás Terrados, Álvaro Miguel-Ortega, Juan Mielgo-Ayuso, Seifeddine Brini, Marco Pernigoni, Daniele Conte, Francesc Cos, Pedro Alcaraz, Roberto Sassi, Pat Viroux, Marko Stojanovic, Enrique Alonso Pérez-Chao, Sergio Ibáñez, Jaime Sampaio, Baris Kocaoglu, Alejandro Vaquera, Anne Delextrat, Julio Calleja-González

**Affiliations:** 1 Department of Physical Education, The Research Center for Sports and Physical Activity, Tel Hai University of Kiryat Shmona in the Galilee, Kiryat Shmona, Israel; 2 Wingate Institute, Levinsky-Wingate Academic College, Netanya, Israel; 3 Sport, Exercise and Human Movement (SEaHM), University of Vic–Central University of Catalonia, Barcelona, Spain; 4 Centre for Studies on Sport and Physical Activity (CEEAF), University of Vic–Central University of Catalonia, Barcelona, Spain; 5 National Institute of Physical Education of Catalonia (INEFC), University of Barcelona, Barcelona, Spain; 6 Sports Performance Department, FC Barcelona, Barcelona, Spain; 7 Football Research Group, Friends’ Club (Club de Amigos, CDA), Buenos Aires, Argentina; 8 Faculty of Health Sciences, European University of the Atlantic, Santander, Spain; 9 Faculty of Kinesiology, University of Zagreb, Zagreb, Croatia; 10 University of Udine, Udine, Italy; 11 Regional Unit of the Institute for Research into Sports Medicine and Health of the Principality of Asturias (ISPA), Oviedo, Spain; 12 Faculty of Education, Alfonso X El Sabio University (UAX), Madrid, Spain; 13 Department of Health Sciences, Faculty of Health Sciences, University of Burgos, Burgos, Spain; 14 Higher Institute of Sport and Physical Education, University of Jendouba, El Kef, Tunisia; 15 Research Unit in Sport, Health, and Movement Sciences, University of Jendouba, El Kef, Tunisia; 16 Department of Coaching Science, Lithuanian Sports University, Kaunas, Lithuania; 17 Department of Movement, Human and Health Sciences, University of Rome “Foro Italico”, Rome, Italy; 18 Institute of Sports Science and Innovation, Lithuanian Sports University, Kaunas, Lithuania; 19 UCAM Research Centre for High-Performance Sport, Catholic University of Murcia, Murcia, Spain; 20 Faculty of Sports Sciences, Catholic University of Murcia, Murcia, Spain; 21 Sports Science Department, FC Internazionale Milano, Milan, Italy; 22 CustoRec, Recovery Cryo Applications Department, Paris, France; 23 Faculty of Sport and Physical Education, University of Novi Sad, Novi Sad, Serbia; 24 Faculty of Sports Sciences, Alfonso X El Sabio University (UAX), Madrid, Spain; 25 Research Group on Training Optimisation and Sports Performance (GOERD), Faculty of Sports Sciences, University of Extremadura, Cáceres, Spain; 26 University of Trás-os-Montes and Alto Douro, Vila Real, Portugal; 27 Department of Orthopaedic Surgery, Faculty of Medicine, Acibadem University, Istanbul, Türkiye; 28 Valoración Funcional e Investigación en el Deporte (VALFIS) Research Group, Institute of Biomedicine (IBIOMED), University of León, León, Spain; 29 Faculty of Sport and Exercise Sciences, University of Worcester, Worcester, United Kingdom; 30 Department of Sport and Health Sciences and Social Work, Oxford Brookes University, Oxford, United Kingdom; 31 Department of Physical Education and Sport, Faculty of Education and Sport, University of the Basque Country (UPV/EHU), Vitoria, Spain

**Keywords:** elite sport, micro-recovery dosing, periodization, psychological detachment, recovery science, staff well-being

## Abstract

Recovery in elite professional sport is now widely understood as a multidimensional process spanning physiological, psychological, cognitive, and social domains, yet recovery models applied in professional clubs and sporting organisations have been slow to reflect this. Most remain reactive and athlete-centred, leaving the full complexity of competitive fatigue inadequately addressed and the equivalent recovery demands on coaching and performance staff entirely unrecognised. No structural framework currently places initiative-taking, calendar-embedded multidimensional recovery as a deliberate component of seasonal planning. Main body. We introduce *micro-recovery dosing* (MRD): proactively planned, pre-scheduled 36-hour multidimensional recovery periods embedded within the competitive season as a proposed starting point at five to eight instances per year, extending to all members of the high-performance organisation. Drawing on neuromuscular recovery kinetics, autonomic science, sleep architecture research, occupational recovery psychology, and complex systems theory, we construct a convergent theoretical rationale for the proposed parameters and distinguish MRD from related constructs. We present a role-differentiated implementation framework with supporting evidence and a discussion of emerging AI-driven monitoring tools. Conclusions. Taken together, the four-domain convergent rationale and the complex systems perspective provide a theoretically coherent basis for MRD’s proposed structure. We argue for a specific organisational transformation: the shift from recovery as an incidental gap between performance demands to an intentionally designed recovery architecture that encompasses athletes and staff alike. The article identifies the active ingredient problem as the central scientific challenge and sets out a structured empirical agenda.

## 1 Introduction

How elite sport understands recovery has changed over the past two decades. Where the field once focused on tissue repair and metabolic restoration, there is now broad consensus that fatigue in professional sport is multidimensional, simultaneously physiological, psychological, cognitive, and social, and that addressing any one domain in isolation leaves the others unresolved (Calleja-González et al., 2018; Kellmann et al., 2018; Altarriba-Bartes et al., 2021; Singh et al., 2021). The practical consequences of accumulated fatigue are real: impaired performance, elevated injury risk, athlete burnout, reduced wellbeing, and shortened careers (Brett et al., 2025; Lambrich and Muehlbauer, 2025). Evidence-based tools exist, from hydrotherapy and targeted nutrition to HRV-guided load management and cryotherapy (Altarriba-Bartes et al., 2021; Sedano and Maroto-Izquierdo, 2025). But uptake remains patchy, and implementation frequently reactive, applied after fatigue becomes visible rather than designed into the season in advance (Altarriba-Bartes et al., 2021; Li et al., 2025).

Less discussed, and, we would argue, equally important, is the recovery of performance staff. Coaches, analysts, physiotherapists, and medical personnel work to the same competitive calendar as their athletes, with nocturnal workloads and no formal recovery provisions (Calleja-González et al., 2021). Poor communication quality between medical staff and head coaches predicts higher injury burden in professional football (Ekstrand et al., 2019); coach burnout impairs decision quality and athlete safety (Goodger et al., 2007; Lundkvist et al., 2012); and deteriorating performance leadership under accumulated fatigue has measurable effects on squad cohesion and tactical consistency (Fletcher and Arnold, 2011; Bentzen et al., 2020). Staff recovery nonetheless scarcely features in sport science frameworks or applied protocols (Calleja-González et al., 2021), the evidence supporting staff-specific recovery is considerably more limited than that for athletes. Future studies should be considered in order to give specific tool for staff members.

This Current Opinion introduces *micro-recovery dosing* (MRD) as a central concept and a structural response to both gaps. It is a theoretical-conceptual paper as a proposed starting point: we draw selectively on cross-disciplinary evidence to advance and justify a testable recovery framework, not to report primary data. We aim to define MRD and distinguish it from existing constructs; to build a multi-domain theoretical rationale for its parameters; to present a role-differentiated implementation guide incorporating emerging AI-driven monitoring tools; and to lay out the scientific limitations and the empirical agenda that follow from them.

## 2 Conceptual positioning: MRD and existing constructs

We define micro-recovery dosing (MRD) as the proactively planned, pre-scheduled integration of structured, multidimensional recovery periods within the elite-sport competitive season, applicable to athletes and all performance staff. The following three features define it: (1) *proactive scheduling*, MRD periods are planned into the season calendar independent of acute post-competition fatigue, not triggered by it; (2) *simultaneous multidimensionality*, each period addresses physiological, cognitive, emotional, and social recovery concurrently; and (3) *organisational universality*, the intervention applies to everyone in the high-performance environment, not only to athletes.

This combination does not describe any existing construct. Active recovery days and passive rest are reactive and athlete-centred, quality depending on unstructured individual choice (Calleja-González et al., 2018; Kellmann et al., 2018). Deloading periods share initiative-taking scheduling but concern only athletes, calibrated to training-load parameters rather than to multidimensional recovery logic (Meeusen et al., 2013; Halson, 2014). Psychological detachment, disengagement from work during non-work time, is the internal experience MRD enables; it is a psychological process, not an organisational intervention (Sonnentag and Fritz, 2007). *Ad hoc* rest days lack strategic timing, a structured framework, and any extension to staff. The distinction we are drawing is not taxonomic tidiness.

The deeper theoretical case for initiative-taking scheduling rests on a fundamental property of complex systems: *hysteresis.* Near tipping points, the path into degraded function differs from the path out. Once a threshold is crossed, restoring baseline requires a disproportionately larger perturbation than the one that caused the breakdown (Balagué et al., 2013; Kiely, 2018). Waiting for visible signs of distress risks acting past that threshold, where recovery is no longer proportionate. MRD prevents approach to tipping points rather than managing their aftermath. The point is that intentionally designed recovery architecture as a proposed starting point rather than a validated prescription, given that does not yet exist in elite sport, and that absence is the problem MRD addresses.

## 3 Discussion

### 3.1 The multidimensional fatigue landscape

Conventional recovery monitoring in elite sport is heavily biased toward physiological biomarkers, and this creates a blind spot. Mental fatigue arising from tactical preparation, media obligations, and the sustained interpersonal intensity of highly demanding elite sport environments increases perceived exertion, reduces time to exhaustion, and elevates internal training load independently of any physical work (Peake et al., 2017; Halperin and Vigotsky, 2024). More subtly, video-analysis sessions and tactical debriefings that coaches classify as “light days” impose directed-attention costs whose downstream effects on training quality and match performance remain inadequately measured. The same applies to staff: coaches, analysts, and medical personnel accumulate cognitive and emotional loads across competition weeks that translate directly into vulnerabilities in decision quality and communication (Goodger et al., 2007; Ekstrand et al., 2019; Calleja-González et al., 2021).

Addressing this requires genuine psychological detachment, not merely physical rest, but the subjective suspension of performance-related cognitive processing (Sonnentag and Fritz, 2007). Athletes and staff can spend nominally free time in continued arousal: monitoring team channels, reviewing footage, processing a result. That state is not recovery, even when no training is happening (Sonnentag and Fritz, 2007). Organisational cultures that reward constant availability make this worse. When being seen to respond quickly is implicitly valued, the cognitive distance required for genuine restoration rarely materialises, even when the calendar says, “day off.” Designing MRD therefore means designing the organisational conditions that permit detachment, not just the schedule that formally enables it.

### 3.2 The 36-hour duration: convergent evidence across four domains

We did not arrive at 36 hours from a single line of evidence. Four domains converge on this window, suggesting it may represent a qualitative threshold, a phase transition between a system under competitive constraint and one genuinely released from it (Balagué et al., 2013). No single domain establishes this; their convergence makes it plausible (**Figure 1**).

**Figure 1:**
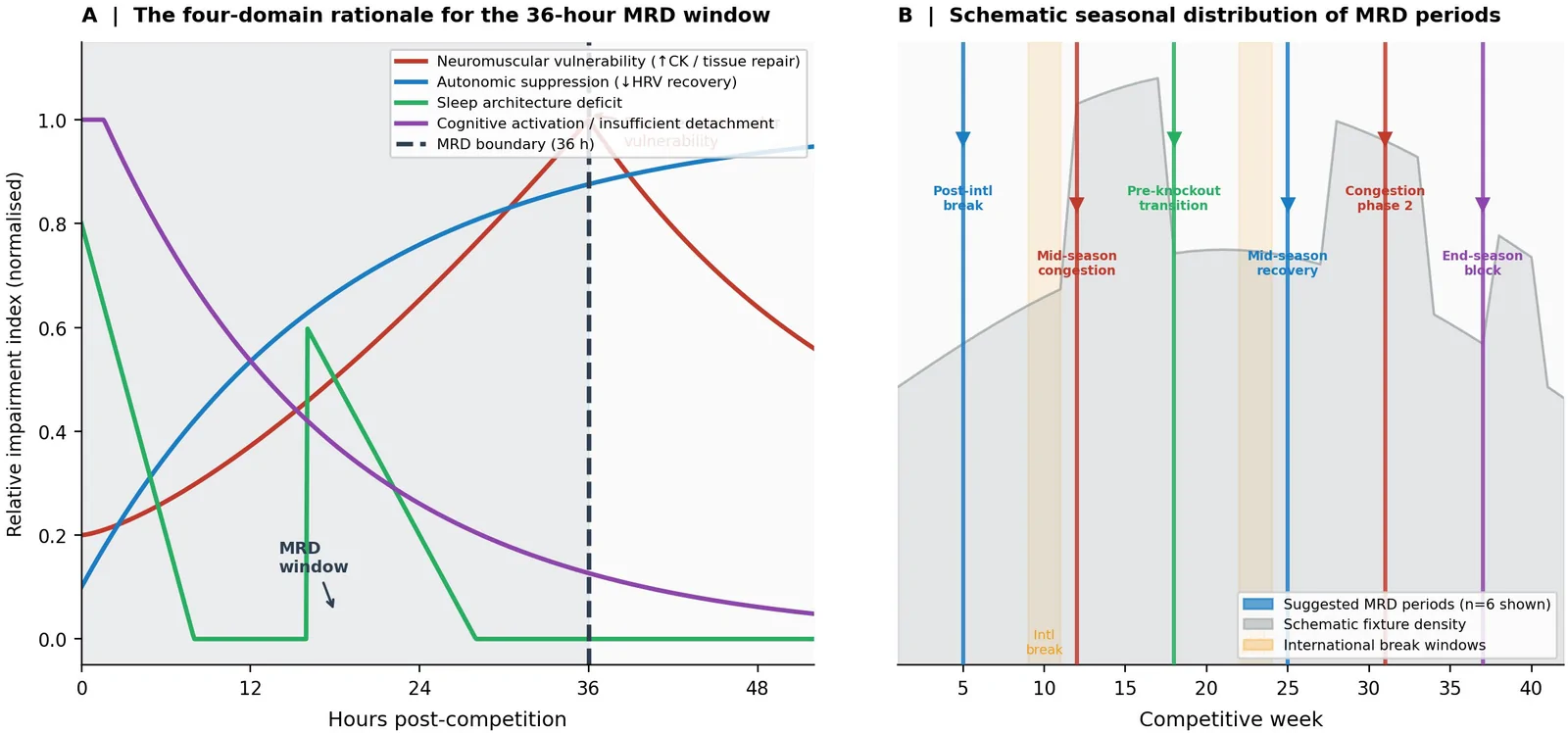
**(A)** Normalized schematic temporal curves for four recovery domains from 0 to 52 hours post-competition. Neuromuscular vulnerability (red) reflects creatine kinase elevation and tissue repair demand, peaking at 36–48 h (Peake et al., 2017). Autonomic suppression (blue) represents HRV deficit recovering over 24–48 h (Nédélec et al., 2012; Buchheit, 2014). Sleep architecture deficit (green) reflects post-competition sleep disruption requiring multiple recovery cycles (Mah et al., 2011; Fullagar et al., 2015). Cognitive activation and insufficient psychological detachment (purple) decline progressively over 36+ hours, with persistent digital connectivity attenuating the rate of decline (Sonnentag and Fritz, 2007). The shaded zone (0–36 h) represents the proposed MRD window; the dashed vertical line marks the 36-hour boundary. Curves are normalised and schematic; exact trajectories vary by individual, sport, and competitive context. **(B)** Schematic seasonal distribution of six MRD periods (colored vertical lines) plotted against fixture density (grey fill) across a 42-week elite team sport season. MRD positions illustrate evidence-informed insertion windows (Meeusen et al., 2013; Halson, 2014; Janse van Rensburg et al., 2021); international break periods are shaded in amber. Specific scheduling requires individual calendar analysis and accumulated load data.

#### 3.2.1 Neuromuscular recovery kinetics

Match play induces muscle damage that peaks, in terms of creatine kinase activity, sarcomeric disruption, and localised inflammatory response, between 24- and 48-hours post-competition (Peake et al., 2017). Resuming high-intensity loading before this window has passed means training into peak structural vulnerability. Thirty-six hours does not guarantee full resolution, but it spans the critical transition.

#### 3.2.2 Autonomic nervous system recovery

HRV-derived cardiac vagal modulation takes 24–48 hours to return to individual baseline after high-intensity match play, with substantial inter-individual variation (Nédélec et al., 2012; Buchheit, 2014). The MRD window places the phase of deepest parasympathetic suppression within the protected period, not at the start of the next training session.

#### 3.2.3 Sleep architecture

Late match finishes, sympathetic arousal, and circadian disruption degrade sleep quality (Fullagar et al., 2015). Sleep extension produces measurable improvements in sprint times, shooting accuracy, and daytime alertness, but benefits accumulate only across multiple consecutive extended nights (Mah et al., 2011). A 36-hour window gives athletes both a restorative sleep episode and an obligation-free morning; an overnight recovery day gives neither.

#### 3.2.4 Psychological detachment

Under conditions of digital connectivity, brief rest periods rarely generate the psychological distance from competitive demands that genuine restoration requires; suppressing work-related thought actively consumes the cognitive resources that recovery is meant to replenish (Sonnentag and Fritz, 2007). High-responsibility roles in elite sport, such as head coaches, team managers, and senior medical staff, face especially persistent cognitive activation. Thirty-six hours allows a progressive transition toward genuine detachment that overnight rest cannot reliably provide.

### 3.3 Five to eight instances per season: periodisation logic and sport-specific feasibility

How often should MRD periods occur? Across the 34–46 competitive weeks of a typical elite team sport season, five to eight instances represent 14-22% of competitive weeks, a proportion broadly consistent with the recovery micro cycle frequencies recommended in periodisation literature to prevent overreaching without compromising fitness (Meeusen et al., 2013; Halson, 2014; Huyghe et al., 2018; Singh et al., 2021). The rhythm this implies is one MRD period every four to six weeks, as a flexible planning guideline, with timing adjusted to the competitive schedule and key competition demands. This frequency is a hypothesis requiring empirical validation rather than an evidence-based recommendation, given that for the best of the author’s knowledge no previous evidence has been reported.

Practical feasibility differs by context. English Premier League clubs contesting around 46–52 fixtures across a 38-week season operate at one game every six to seven days; an MRD period every four to six weeks requires no structural change to competitive scheduling, only that an existing gap be used deliberately. In elite rugby union, where Premiership seasons encompass 20–24 fixtures over 30 weeks, the post-match recovery micro cycle already provides structural support for MRD integration without schedule modification.

Evidence-informed insertion windows include post-international break returns, mid-season congestion transitions, post-knockout elimination rounds, and post-transcontinental travel recoveries (Janse van Rensburg et al., 2021). These are starting points, not rules; specific scheduling requires individual calendar analysis and accumulated load data from across the organisation. We emphasise again that 36 hours and five to eight instances are proposals for experimental investigation. They are not validated optima.

### 3.4 A complex systems perspective: towards a mechanistic framework

The four-domain rationale treats each recovery domain as an independent process and the athlete as a set of parallel restoration systems. Complex adaptive systems theory, and specifically the constraints-led approach, the framework in which behaviour emerges from the interaction of organism, task, and environmental constraints (Balagué et al., 2013; Pol et al., 2020), offers a materially different framing.

Complex adaptive systems theory, as applied to sport, reframes the athlete as a coupled psychobiological system in which physiological, psychological, and social processes are not parallel but deeply interdependent (Balagué et al., 2013; Pol et al., 2020). Match play does not simply perturb individual subsystems; it disrupts the coordination relationships between them. And that coordination, not any single subsystem, is what constitutes functional readiness. Restoring neuromuscular capacity while autonomic suppression and sleep debt persist does not return the athlete to a recovered state; it returns them to a state in which some parameters have normalised while the system remains destabilised (Balagué et al., 2013; Pol et al., 2020). For the system to re-organise around full functional readiness, it must be released simultaneously from the multiple constraints preventing self-organisation (Kiely, 2018). A single ice bath, a single sleep episode, or a nature walk addresses one constraint. MRD addresses several simultaneously. The same logic extends to the athlete-staff system: a fatigued coach constitutes an additional constraint on the athlete, one that individual athlete recovery alone cannot remove (Fletcher and Arnold, 2011; Ekstrand et al., 2019).

From this perspective, MRD is a structured removal of simultaneous constraints across different timescales- physical, cognitive, social, environmental- creating the conditions under which cross-subsystem re-coupling can emerge without further performance-directed intervention (Pol et al., 2020). Crucially, this reframing does not dissolve the active ingredient problem; it redefines the experimental question. The question is no longer which component produces the measurable effect. It is whether simultaneous constraint-removal generates a systemic recovery response that cannot be replicated by removing any single constraint alone. That is a testable hypothesis, and it implies the factorial designs described in Section 6.

The framework directly justifies MRD’s three defining features. Initiative-taking scheduling ensures constraint-removal occurs at peak perturbation; not reactively once fatigue is visible. Organisational universality extends removal to include social and professional constraints that individual rest days leave intact. And structured monitoring provides the feedback loop needed to confirm the system has re-stabilised before competitive loading resumes (Balagué et al., 2013; Kiely, 2018).

## 4 Practical implementation framework

Six evidence-grounded components constitute the MRD period, each with its empirical basis noted to support component-isolation in future research. Sleep extension targeting ≥8–9 hours with sufficiently early bedtimes and early obligations removed is the most directly supported: multiple consecutive extended nights improve sprint performance, shooting accuracy, and alertness in elite athletes (Mah et al., 2011). Digital disconnection from team channels, tactical material, and performance media prevents the cognitive activation that attenuates restoration (Sonnentag and Fritz, 2007). Positive social engagement outside the team environment buffers occupational stress through direct wellbeing effects and physiological stress-buffering pathways (Cohen and Wills, 1985). Nature exposure restores directed-attention circuits and reduces stress biomarkers relative to urban conditions (Kaplan, 1995; Hartig et al., 2003; Berman et al., 2008). Autonomy over activity selection is a design principle: prescribing specific activities during an MRD period is counterproductive (Calleja-González et al., 2018). Restorative nutrition should reflect reduced energy expenditure while sustaining protein intake for tissue repair (Cheng et al., 2025).

How these components are emphasised depends on role. A starting player managing significant eccentric muscle damage and post-match cognitive overload needs something quite different from a reserve athlete processing the emotional weight of limited playing time, or an injured player navigating pain and identity disruption, or a head coach whose decision-making has been under sustained load for three days. **Table 1** sets out a role-differentiated framework grounded in the same evidence base; it is guidance, not protocol, and individual adaptation is expected.

**Table 1:** Role-differentiated micro-recovery dosing (MRD) strategies.

Role	Primary recovery demand	Priority domains	Evidence-informed MRD activities
Starting Athletes (High-Minute)	Eccentric musculoskeletal load (Peake et al., 2017) Late-match sleep disruption (Fullagar et al., 2015) Cognitive and emotional fatigue	Tissue repair (Peake et al., 2017) Autonomic restoration (Nédélec et al., 2012; Buchheit, 2014) Sleep extension (Mah et al., 2011) Detachment (Sonnentag and Fritz, 2007)	Sleep-in: ≥8–9 h; nap optional (Mah et al., 2011) 20–30 min outdoor walk, ART: natural environments restore directed attention (Kaplan, 1995; Berman et al., 2008) Digital detox until noon (Sonnentag and Fritz, 2007) Social time with family or close friends (Cohen and Wills, 1985)
Reserve Athletes	Emotional load from role uncertainty and identity stress Physiological under-stimulation relative to squad	Emotional regulation Moderate activation Validation	25–40 min recreational activity away from the club facility (Example can be adapted to the individual and organizational context) Informal practitioner check-in preferred over digital questionnaire (Saward et al., 2024) Cognitive reset: reading, journaling, or mindfulness (Sonnentag and Fritz, 2007) Role-clarity discussion scheduled for next training session
Injured Athletes	Pain and immobility (Peake et al., 2017) Identity disruption from team isolation Rehabilitation uncertainty	Pain modulation Emotional support Social belonging	Pool walking or hydrotherapy if clinically indicated (Altarriba-Bartes et al., 2021) Slow outdoor walk or nature exposure to reduce rumination (Kaplan, 1995) Social contact outside the clinical environment (Cohen and Wills, 1985) All performance-related meetings suspended during MRD period
Head Coach	Extreme decision fatigue (Fletcher and Arnold, 2011; Calleja-González et al., 2021) Post-match media obligations Sleep restriction from post-game analysis	Cognitive decompression (Kaplan, 1995; Berman et al., 2008) Sleep extension (Mah et al., 2011) Psychological detachment (Sonnentag and Fritz, 2007)	No tactical review until afternoon of Day 2 60–90 min outdoor walk, nature exposure restores executive attention (Hartig et al., 2003; Berman et al., 2008) Screen-free and club-silent morning (Sonnentag and Fritz, 2007) Leisure activity explicitly excluding decision demands
Support Staff (Coaching, S&C, Medical, Analysis)	Sustained cognitive and physical load (Calleja-González et al., 2021) Constant-availability expectation Screen fatigue and social saturation	Rest and disconnection (Sonnentag and Fritz, 2007) Musculoskeletal recovery (Altarriba-Bartes et al., 2021) Social recharge (Cohen and Wills, 1985)	Minimum 8–12 h protected no-contact window (Sonnentag and Fritz, 2007) Personal light physical activity (Altarriba-Bartes et al., 2021) Unstructured family or social time (Cohen and Wills, 1985) Screen-free morning for analysts; analytical work deferred to afternoon of Day 2
Performance and Operations Management	High-stakes decision pressure Multi-stakeholder communication load Emotional burden of competitive responsibility	Cognitive distancing Perspective building	No roster or contract discussions until afternoon of Day 2 Extended outdoor or social time with family (Cohen and Wills, 1985; Kaplan, 1995) Mindfulness or reflective practice (Sonnentag and Fritz, 2007) Complete disengagement from competitive communications Digital-disconnection periods (Example can be adapted to the individual and organizational context)

Evidence bases for specific activities indicated in brackets. Individual adaptation is expected and encouraged. ART, Attention Restoration Theory.

### 4.1 Monitoring integration

Deciding when to schedule an MRD period requires more than a calendar heuristic. A practical monitoring workflow combines brief validated wellness instruments, the Short Recovery and Stress Scale or session-RPE (Kellmann et al., 2018), with informal practitioner communication, which consistently demonstrates greater ecological validity than digital questionnaire completion when delivered with genuine relational intent (Saw et al., 2016; Saward et al., 2024). For objective monitoring, resting morning HRV suppression below an individual’s established baseline over three or more consecutive mornings is a reliable trigger (Nédélec et al., 2012; Buchheit, 2014). From a complex systems perspective, what matters most is not mean suppression but reduction in HRV complexity, the loss of adaptive variability that signals a healthy system near criticality (Balagué et al., 2013; Buchheit, 2014). A rigidly monotonic autonomic response indicates a system approaching a tipping point. MRD monitoring should deal with this complexity reduction, not only mean-level departures. No single indicator overrides clinical judgement.

AI-driven augmentation of this workflow is already technically feasible. Platforms that integrate wearable sensor outputs, HRV metrics, GPS-derived load data, and wellness questionnaire responses in real time are increasingly common in professional sport environments, and AI has demonstrated utility for synthesising multimodal signals that no single instrument captures adequately (Mateus et al., 2025). Applied to MRD, machine learning models trained on longitudinal multimodal data could in principle identify converging multidimensional fatigue signatures, declining HRV, suppressed wellness scores, behavioural indicators of insufficient off-training recovery, and flag scheduling windows with sensitivity that calendar-based rules cannot match. Whether this adds clinical value over expert practitioner judgement is itself a testable question, and one worth asking.

## 5 Limitations

These limitations are not routine methodological caveats. They are substantive scientific constraints that determine what this article can and cannot claim.

### 5.1 The active ingredient problem, the central scientific challenge

MRD bundles sleep extension, psychological detachment, social engagement, nature exposure, nutritional support, and activity autonomy into a single 36-hour window. If a future trial shows MRD works, we will not immediately know why. The benefit could come from any single component, from their interaction, from the duration alone, from the initiative-taking scheduling and its expectancy effects, or from the load reduction that any protected rest period provides. The complex systems framing in Section 3.4 offers a theoretical reason to expect that simultaneous constraint-removal produces effects exceeding the sum of its parts, but this is a hypothesis, not evidence. The most uncomfortable version of the problem is this: sleep extension alone, on current evidence, would be expected to yield measurable performance improvements (Mah et al., 2011). Without a factorial design, a positive MRD outcome cannot be attributed to anything more specific. This is why the paper must be read as a proposal for investigation rather than as a protocol.

### 5.2 Absence of direct empirical validation

The proposed parameters, 36 hours, five to eight instances, come from theoretical synthesis across adjacent domains, not from any study of MRD as a distinct intervention. They are starting points for experimental research. We do not claim the evidence base that clinical or performance management guidelines would require.

### 5.3 Institutional barriers

Broadcasting rights, fixture scheduling, contractual obligations, and governance structures constrain calendar flexibility in ways this article has not addressed. Implementing five to eight collectively protected 36-hour recovery periods per season is genuinely difficult in environments with 80+ annual fixtures and multiple stakeholders. Implementation science research, who holds the leverage points, and under what conditions adoption is feasible, is not a secondary priority. It may be the prerequisite for efficacy research.

### 5.4 Individual versus organisational implementation

For most components, individual implementation is possible without institutional support. But psychological detachment, what distinguishes MRD from ordinary rest, is modulated by organisational norms: when constant availability is implicitly rewarded, people report attenuated detachment quality during nominally free time (Sonnentag and Fritz, 2007). The individual who sleeps in and spends the afternoon with family captures the physiological and sleep components. More Professional & Natural: They will not fully achieve the cognitive detachment that only an organization-wide removal of constraints can provide. Individual education and institutional culture change are complementary, not alternatives.

### 5.5 Population specificity and measurement validity

The evidence base skews heavily toward adult male professional team sport; how MRD parameters should change across different sexes, age groups, career stages, and sport types is an open empirical question (Mason et al., 2025). The monitoring tools described were not developed for MRD-specific applications, and instruments sensitive to detachment depth and integrated multidimensional recovery quality within a 36-hour window do not yet exist. In fact, a protected 36-hour period may be realistic during some phases of a season but difficult during congested competition schedules, tournaments, or international travels.

## 6 Future research directions

The most important experiment to run is a randomised crossover trial with four conditions: full 36-hour MRD; sleep extension alone; sleep extension combined with psychological detachment but without the remaining components; and standard post-competition recovery. This design directly addresses the active ingredient problem and generates the first empirical test of the 36-hour duration parameter itself. Primary outcomes should include neuromuscular performance indices, morning HRV trajectories, standardised directed-attention tests, the Recovery Experience Questionnaire (Sonnentag and Fritz, 2007), and session-RPE from the training session immediately after each condition.

Season-long cohort studies comparing teams with and without MRD would address what individual crossover trials cannot: the cumulative effects on injury incidence, match availability, wellness trajectories, and burnout prevalence across a full competitive season.

Staff-focused research is urgently needed. Occupational load, recovery quality, and decision performance in coaching and support staff remain entirely unstudied in the elite sport context (Fletcher and Arnold, 2011; Lundkvist et al., 2012), and validated measurement tools for this population are lacking.

Individual moderators of MRD response, recovery phenotype, sleep architecture, detachment style, and social network characteristics are likely to be important; the role-category framework in **Table 1** is a starting approximation. AI-driven scheduling support, integrating multimodal longitudinal data to personalise both timing and component weighting of MRD periods (Mateus et al., 2025), offers a technically feasible pathway toward individually calibrated recovery prescriptions that warrants dedicated investigation.

Finally, implementation science. Understanding which stakeholders hold the leverage points, what the perceived and real barriers to adoption are, and how MRD can be integrated within the institutional and commercial constraints of elite professional sport is as important as establishing physiological efficacy. Neither strand of research alone is sufficient.

## 7 Conclusions

Elite professional sport imposes multidimensional demands that current recovery practice does not adequately meet. The proposal we advance here is structural: MRD places recovery, for athletes and for staff, as a deliberately designed component of performance architecture rather than an incidental gap between fixtures.

The case for MRD rests on four convergent evidence domains- neuromuscular kinetics, autonomic science, sleep architecture, and occupational recovery psychology- and on a complex system framing that, to our knowledge, has not previously been applied to the design of structured recovery periods in elite sport. From that perspective, MRD is not a bundle of independent recovery components. It is a structured removal of simultaneous constraints- physical, cognitive, social, environmental- under which systemic recovery can emerge without further performance-directed intervention. That reframing redefines the experimental question: not “which component works?” but “does simultaneous constraint-removal produce a systemic effect no single component replicates?”, a question with a testable answer and one the field should now pursue.

The limitations in Section 5 are not arguments against MRD. They define the research agenda (the suggested comparison of full MRD with sleep extension alone, partial MRD, and usual recovery is a useful way to determine whether MRD provides benefits beyond individual recovery components) it demands: factorial trials, season-long cohort studies, staff-focused measurement, and implementation science. What they describe, collectively, is the distance between a theoretically grounded proposal and a validated protocol. Closing that distance is the work ahead. What we propose here is a starting architecture, and a reason to begin.

## Data Availability

The original contributions presented in the study are included in the article/supplementary material. Further inquiries can be directed to the corresponding author.
